# Long-term exposure to air pollution and risk of Sarcopenia in adult residents of Taiwan: a nationwide retrospective cohort study

**DOI:** 10.1186/s12889-023-17091-8

**Published:** 2023-11-06

**Authors:** Ssu-Wen Chen, Chih-Ying Lin, Chiu-Ying Chen, Cheng-Li Lin, Tsai-Ling Hsieh, Fuu-Jen Tsai, Kuang-Hsi Chang

**Affiliations:** 1 Department of Family Medicine, Tungs’ Taichung MetroHarbor Hospital, Taichung, 435 Taiwan; 2 Department of Public Health, China Medical University, Taichung, 404 Taiwan; 3 Management Office for Health Data, China Medical University Hospital, Taichung, 404 Taiwan; 4 College of Medicine, China Medical University, Taichung, 404 Taiwan; 5 Department of Medical Research, Tungs’ Taichung MetroHarbor Hospital, Taichung, 435 Taiwan; 6 Department of Otolaryngology, Tungs’ Taichung MetroHarbor Hospital, Taichung, 435 Taiwan; 7 School of Chinese Medicine, College of Chinese Medicine, China Medical University, Taichung, 404 Taiwan; 8 Department of Medical Research, China Medical University Hospital, China Medical University, Taichung, 404 Taiwan; 9 Division of Medical Genetics, China Medical University Children’s Hospital, Taichung, 404 Taiwan; 10 Department of Biotechnology and Bioinformatics, Asia University, Taichung, 413 Taiwan; 11 Center for General Education, China Medical University, Taichung, 404 Taiwan; 12 General Education Center, Nursing and Management, Jen-Teh Junior College of Medicine, Miaoli, 356 Taiwan

**Keywords:** Air pollution, Sarcopenia, Adult residents, Cohort study

## Abstract

**Background:**

Sarcopenia is an age-related, multifactorial syndrome. Previous studies have shown that air pollutants are associated with inflammation and oxidative stress. However, the association between long-term exposure to air pollution and sarcopenia is not completely understood.

**Methods:**

The Taiwan National Health Research Database (NHIRD) contains medical records of almost all Taiwanese residents. Daily air pollution data collected by the Taiwan Environmental Protection Agency was used to analyze concentrations of sulfur oxide (SO_2_), carbon monoxide (CO), nitrogen monoxide (NO), nitrogen dioxide (NO_2_), and particulate matter (PM_2.5_, PM_10_). The databases were merged according to the insurants’ living area and the location of the air quality monitoring station. We categorized the pollutants into quartiles (Q1, Q2, Q3, and Q4).

**Results:**

Our study population consisted of 286,044 patients, among whom 54.9% were female and 45.1% were male. Compared to Q1 levels of pollutants, Q4 levels of SO_2_ (adjusted hazard ratio [aHR] = 8.43; 95% confidence interval [CI] = 7.84, 9.07); CO (aHR = 3.03; 95%CI = 2.83, 3.25); NO (aHR = 3.47; 95%CI = 3.23, 3.73); NO_2_ (aHR = 3.72; 95%CI = 3.48, 3.98); PM_2.5_ (aHR = 21.9; 95% CI = 19.7, 24.5) and PM_10_ (aHR = 15.6; 95%CI = 14.1, 17.4) increased risk of sarcopenia.

**Conclusions:**

Our findings indicated a significantly increased risk of sarcopenia in both male and female residents exposed to high levels of air pollutants.

## Introduction

Over the past decade, air pollution has become an important public health issue in industrialized countries [1, 2]. Long-term exposure triggers tissue-specific inflammation and increases oxidative stress, resulting in an increased risk of carcinoma, cardiovascular diseases, cerebrovascular diseases, neurodegenerative diseases, and osteoporosis, among others [3–7]. Sarcopenia is an age-related, multifactorial syndrome caused by low physical activity, poor nutritional consumption, changes in sex hormones, and oxidative stress, [8–12] primarily presenting as a loss of skeletal muscle mass and strength [13]. Thus, it often causes fractures, functional decline, frailty, and mortality, especially in patients with solid tumors, kidney dysfunction, diabetes, and cirrhosis [14–17]. Nevertheless, its etiology and pathogenesis are yet to be elucidated [18]. Inflammatory markers and oxidative stress have been proven to be associated with the onset of sarcopenia [19–21]. Past sarcopenia-related research was most frequently conducted on middle-aged or elderly people [22–24]. In a cross-sectional study, for each unit Increase in air pollutants, the sarcopenia risks were increased by 11.1%, 4.3%, 22.6%, and 9.3% in PM_2.5_, PM_10_, SO_2,_ and O_3_, respectively [22]. Zhihan et al. observed an increased risk of probable sarcopenia with each interquartile range increment in the following air pollutants: PM_2.5_ (Odds Ratio (OR): 1.06; 95% Confidence Interval (CI): 1.04, 1.07); PM_10_ (OR: 1.15; 95% CI: 1.13, 1.17); PM coarse (O: 1.02; 95% CI: 1.01, 1.03); PM_2.5_ (OR: 1.08; 95% CI: 1.07, 1.10); NO2 (OR: 1.12; 95% CI: 1.10, 1.14); NOx (OR: 1.06; 95% CI: 1.05, 1.08) in older people [25].

However, > 10% of young adults of most ethnicities have been diagnosed with sarcopenia [26]. However, air is an indispensable necessity for life. It is not like hormones, nutritional status, or activity that changes due to differences in age and gender. Thus, we conducted this nationwide retrospective cohort study of Taiwanese residents aged > 18 years to evaluate the risk of sarcopenia associated with long-term exposure to air pollution.

## Methods

### Data source

The Taiwan National Health Research Database (NHIRD) contains the medical records of almost all Taiwanese residents. Patient demographic information, except for private data, time of diagnosis, and use of medicine, was extracted from the NHIRD. Disease coding followed the International Classification of Diseases, Ninth Revision, Tenth Revision, and Clinical Modification (ICD-9-CM and ICD-10-CM). In this study, we used the Longitudinal Generation Tracking Database, a subset of data from the NHIRD with a population of 2 million. For air pollution data, we used daily data from the Taiwan Environmental Protection Agency (EPA). The Taiwan EPA, Executive Yuan has established 74 ambient air quality monitoring stations (AQMS) on Taiwan’s main island, with locations based on population density. In this study, we obtained air pollutant levels from 31, 15, 23 and 5 AQMS located in northern, central, southern, and eastern Taiwan respectively. We analyzed temperature, humidity, sulfur oxide (SO_2_), carbon monoxide (CO), nitrogen monoxide (NO), nitrogen dioxide (NO_2_), and particulate matter (PM_2.5_, PM_10_). The databases were merged according to insurants’ living area and location of the air quality monitoring station.

This study was approved by the Institutional Review Board of the China Medical University Hospital Research Ethics Committee (CMUH109-REC2-031).

### Study population, outcome, and comorbidities

We observed that the population aged > 18 years in 2003 lived in an area with air quality monitoring stations and had no history of sarcopenia before 2003. Sarcopenia (ICD-9-CM: 728.2; ICD-10-CM: M62.84) was defined as two or more outpatient diagnoses or one admission record. The study period began in 2003 and ended in 2016. Residents with missing data on living areas, medical records, and pollutant levels were excluded, as were those previously diagnosed with sarcopenia before the baseline.

The annual average amount of air pollutants to which each subject was exposed during the follow-up period was calculated: the sum of the pollutant concentrations for the days of exposure is divided by the number of days of exposure. The quartile of each air pollutant was as follows: SO_2_ (Q1: < 2.63 ppb; Q2: 2.63–3.21 ppb; Q3: 3.21–3.54 ppb; Q4: > 3.54 ppb); CO (Q1: < 0.37 ppm; Q2: 0.37–0.45 ppm; Q3: 0.45–0.55 ppm, Q4: > 0.55 ppm); NO (Q1: < 2.56 ppm; Q2: 2.56–4.05 ppm; Q3: 4.05–6.12 ppm; Q4: > 6.12 ppm); NO_2_ (Q1: < 16.1 ppm; Q2: 16.1–21.2 ppm; Q3: 21.2–26.9 ppm; Q4: > 26.9 ppm); PM_2.5_ (Q1: < 17.0 µg/m^3^; Q2: 17.0–18.4 µg/m^3^; Q3: 18.4–26.3 µg/m^3^; Q4: > 26.3 µg/m^3^); and PM_10_ (Q1: < 34.8 µg/m^3^; Q2: 34.8–39.9 µg/m^3^; Q3: 39.9-–49.7 µg/m^3^; Q4: > 49.7 µg/m^3^). Related comorbidities, such as alcohol abuse/dependence, tobacco abuse/dependence, chronic obstructive pulmonary disease (COPD), asthma, obesity, osteoporosis, and estrogen supplementation, were considered covariates.

### Statistical analysis

The variables of sex, urbanization level, comorbidities, medication, and sarcopenia were presented as counts and percentages. Urbanization was defined using criteria that included population density (people per square kilometer), the proportion of individuals with a college-level education or higher, the proportion of people aged 65 years and older, the proportion of agricultural workers, and the density of physicians per 100,000 residents [27]. Variables related to environmental factors were expressed as means and standard deviations. The incidence rate ratio (IRR) was computed using Poisson regression.

Alcohol abuse, tobacco abuse, COPD, and asthma were related to smoking status and alcohol drinking patterns [28–34]. Obesity, osteoporosis and estrogen were also associated with the development of sarcopenia [8–12, 35]. To control for these confounders, the multivariable Cox proportional models were used to estimate the adjusted hazard ratio (aHR) to reveal the risk of sarcopenia with air pollution exposure in Taiwan residents. We also performed multivariable Cox proportional models with stratification to clarify the association between air pollution and the risk of sarcopenia development. Analyses were performed using SAS (version 9.4; SAS Institute Inc., Cary, NC, USA). Statistical significance was set at p < 0.05.

## Results

Our study population comprised 286,044 patients, of whom 54.9% were female and 45.1% were male. The mean age was 41.0 (± 16.2) years, and the high-level urbanization area was the dominant group. A total of 12.6% of patients had comorbidities such as COPD, and 11.6% had previously developed asthma. Approximately 29.4% of participants took estrogen supplements. As shown in Table 1, the yearly average values of SO_2_, CO, NO, NO_2_, PM_2.5_, and PM_10_ that participants were exposed to within the follow-up period were 3.29 ppb (± 1.08), 0.49 ppm (± 0.19), 6.47 ppm (± 6.94), 23.4 ppm (± 11.7), 21.0 µg/m^3^ (± 6.19) and 43.20 µg/m^3^ (± 11.9), respectively. A total of 8,904 participants developed sarcopenia, and the mean follow-up time was 12.4 ± 2.20 years.

**Table 1:** Baseline demographics and air pollutant exposure by yearly average concentration in Taiwan, 2003–2016 (N = 286,044)

Covariates		n	%
Gender	Female	157,103	54.9
	Male	128,941	45.1
Age, years	mean, SD	41.0	16.2
Urbanization level^†^	1 (highest)	171,442	59.9
	2	93,475	32.7
	3	18,354	6.42
	4 (lowest)	2773	0.97
Comorbidity			
Alcohol abuse/dependence		4917	1.72
Tobacco abuse/dependence		26	0.01
COPD		36,002	12.6
Asthma		33,129	11.6
Obesity		5330	1.86
Osteoporosis		26,461	9.25
Estrogen supplement		84,134	29.4
Exposure of air pollutants			
SO_2_ level (yearly average, ppb)	mean, SD	3.29	1.08
CO level (yearly average, ppm)	mean, SD	0.49	0.19
NO level (yearly average, ppm)	mean, SD	6.47	6.94
NO_X_ level (yearly average, ppm)	mean, SD	23.4	11.7
PM_2.5_ (yearly average, µg/m^3^)	mean, SD	21.0	6.19
PM_10_ (yearly average, µg/m^3^)	mean, SD	43.2	11.9
Outcome			
Sarcopenia		8094	2.83
Follow-up time, years	mean, SD	12.4	2.20

^†^Urbanization level was categorized into four levels by population density of the residential area, with level 1 being the most urbanized and level 4 being the least urbanized

CO, carbon monoxide; COPD, chronic obstructive pulmonary disease; NO, nitric oxide; NO_2_, nitrogen dioxide; PM, particulate matter; Q, quartile; SO_2_, sulfur dioxide; SD, standard deviation

Table 2 shows the relationship between air pollutants and sarcopenia using the IRR. We divided the concentration of pollutants into four quartiles. Considering Q1 levels of each pollutant as the reference, the IRR of sarcopenia in Q2 levels of SO_2_, CO, NO, NO_2_, and PM_2.5_ was 1.37 (95% confidence interval [CI] = 1.25, 1.50), 1.29 (95%CI = 1.19, 1.40), 1.53 (95%CI = 1.42, 1.66), 1.50 (95%CI = 1.39, 1.61), and 0.18 (95%CI = 0.13, 0.25), respectively. Patients exposed to Q3 levels of CO (IRR = 2.00; 95%CI = 1.86, 2.16), NO (IRR = 1.86; 95%CI = 1.72, 2.01), PM_2.5_ (IRR = 4.88; 95%CI = 4.35, 5.49), and PM_10_ (IRR = 5.93; 95%CI = 5.32, 6.61) had an increased rate of sarcopenia. Q4 levels of all pollutants were related to sarcopenia (SO_2_: IRR = 8.74, 95%CI = 8.13, 9.40; CO: IRR = 2.88, 95%CI = 2.69, 3.08; NO: IRR = 3.33, 95%CI = 3.10, 3.57; NO_2_: IRR = 3.63, 95%CI = 3.40, 3.88; PM_2.5_: IRR = 23.0; 95%CI = 20.6, 25.7; PM_10_: IRR = 16.2, 95%CI = 14.6, 18.0). In women, the association between air pollutants and sarcopenia was similar to that observed in the entire population. However, the IRR of sarcopenia in Q3 levels of NO_2_ relative to Q1 levels was 1.23 (95%CI = 1.09, 1.38) in men.

**Table 2:** Incidence and incidence rate ratio of sarcopenia for the four levels of pollutant exposure

Pollutant levels		All				Males				Females			
		Event	IR	IRR	(95%CI)	Event	IR	IRR	(95%CI)	Event	IR	IRR	(95%CI)
SO_2_ (ppb)	Q1, < 2.63	846	0.90			319	0.77			527	1.00		
	Q2, 2.63–3.21	1039	1.24	1.37	(1.25, 1.50)	446	1.19	1.53	(1.33, 1.77)	593	1.28	1.28	(1.13, 1.43)
	Q3, 3.21–3.54	984	0.89	0.99	(0.90, 1.08)	369	0.76	0.98	(0.85, 1.14)	615	0.99	0.99	(0.88, 1.11)
	Q4, > 3.54	5225	8.05	8.74	(8.13, 9.40)	2181	7.11	9.02	(8.02, 10.1)	3044	8.88	8.68	(7.92, 9.52)
CO (ppm)	Q1, < 0.37	1075	1.25			429	1.08			646	1.41		
	Q2, 0.37–0.45	1472	1.62	1.29	(1.19, 1.40)	576	1.43	1.33	(1.17, 1.51)	896	1.76	1.25	(1.13, 1.39)
	Q3, 0.45–0.55	1987	2.52	2.00	(1.86, 2.16)	818	2.33	2.15	(1.91, 2.42)	1169	2.67	1.90	(1.72, 2.09)
	Q4, > 0.55	3557	3.63	2.88	(2.69, 3.08)	1490	3.46	3.19	(2.87, 3.55)	2067	3.75	2.66	(2.43, 2.90)
NO (ppm)	Q1, < 2.56	958	1.13			362	0.95			596	1.28		
	Q2, 2.56–4.05	1538	1.74	1.53	(1.42, 1.66)	622	1.55	1.62	(1.42, 1.85)	916	1.91	1.48	(1.34, 1.64)
	Q3, 4.05–6.12	1619	2.11	1.86	(1.72, 2.01)	684	1.99	2.09	(1.84, 2.37)	935	2.21	1.72	(1.55, 1.90)
	Q4, > 6.12	3979	3.80	3.33	(3.10, 3.57)	1647	3.61	3.77	(3.36, 4.22)	2332	3.95	3.06	(2.79, 3.34)
NO_2_ (ppm)	Q1, < 16.1	1178	1.37			444	1.12			734	1.57		
	Q2, 16.1–21.2	1745	2.05	1.50	(1.39, 1.61)	707	1.85	1.64	(1.46, 1.85)	1038	2.22	1.41	(1.28, 1.54)
	Q3, 21.2–26.9	1599	1.43	1.05	(0.97, 1.13)	675	1.38	1.23	(1.09, 1.38)	924	1.47	0.94	(0.85, 1.03)
	Q4, > 26.9	3572	5.03	3.63	(3.40, 3.88)	1489	4.71	4.14	(3.72, 4.60)	2083	5.29	3.32	(3.05, 3.61)
PM_2.5_ (µg/m^3^)	Q1, < 17.0	337	0.32			123	0.27			214	0.36		
	Q2, 17.0–18.4	38	0.06	0.18	(0.13, 0.25)	15	0.05	0.20	(0.12, 0.34)	23	0.06	0.17	(0.11, 0.26)
	Q3, 18.4–26.3	1752	1.60	4.88	(4.35, 5.49)	746	1.48	5.41	(4.47, 6.55)	1006	1.69	4.60	(3.97, 5.34)
	Q4, > 26.3	5765	7.61	23.0	(20.6, 25.7)	2347	6.67	24.1	(20.1, 28.9)	3418	8.42	22.6	(19.7, 26.0)
PM_10_ (µg/m^3^)	Q1, < 34.8	381	0.40			138	0.34			243	0.45		
	Q2, 34.8–39.9	330	0.37	0.92	(0.79, 1.06)	128	0.33	0.96	(0.75, 1.22)	202	0.40	0.89	(0.74, 1.08)
	Q3, 39.9–49.7	2223	2.41	5.93	(5.32, 6.61)	956	2.25	6.54	(5.47, 7.82)	1267	2.55	5.63	(4.91, 6.46)
	Q4, > 49.7	5160	6.67	16.2	(14.6, 18.0)	2093	5.83	16.8	(14.2, 20.0)	3067	7.39	16.1	(14.2, 18.4)

CI, confidence interval; CO, carbon monoxide; IR, incidence rate (per 1000 person-years); IRR, incidence rate ratio; NO, nitric oxide; NO_2_, nitrogen dioxide; PM, particulate matter; Q, quartile; SO_2_, sulfur dioxide

Based on the Cox proportional hazards model (Table 3), the level of pollutants was directly proportional to the risk of sarcopenia. Similarly, compared to Q1 levels of pollutants, Q2 levels of SO_2_ (aHR = 1.40; 95% CI = 1.28, 1.54); CO (aHR = 1.33; 95%CI = 1.23, 1.44); NO (aHR = 1.53; 95%CI = 1.41, 1.66); and NO_2_ (aHR = 1.51; 95%CI = 1.40, 1.63) increased the risk of sarcopenia, but Q2 levels of PM_2.5_ (aHR = 0.18; 95%CI = 0.13, 0.26) did not. In addition to SO_2_, Q3 levels of pollutants increased the incidence of sarcopenia by 2.08 times (95%CI = 1.93, 2.24) for CO, 1.92 times (95%CI = 1.77, 2.08) for NO, 1.12 times (95% CI = 1.04, 1.21) for NO_2_, 4.82 times (95%CI = 4.29, 5.42) for PM_2.5_, and 5.94 times (95% CI = 5.33, 6.63) for PM_10_. Regarding Q4 levels of pollutants, PM_2.5_ showed the highest aHR of sarcopenia (21.9; 95% CI = 19.7, 24.5), followed by PM_10_ (aHR = 15.6; 95%CI = 14.1, 17.4), and SO_2_ (aHR = 8.43; 95%CI = 7.84, 9.07). Pollutants in Q4 levels of CO (aHR = 3.03; 95%CI = 2.83, 3.25); NO (aHR = 3.47; 95%CI = 3.23, 3.73); and NO_2_ (aHR = 3.72; 95%CI = 3.48, 3.98) increased the risk of sarcopenia more than threefold. The relationship between pollutant levels and sarcopenia remained the same in both men and women.

**Table 3:** Adjusted hazard ratios of sarcopenia in quartiles 2, 3, and 4 compared to that in quartile 1

Pollutant levels		All		Males		Females	
		aHR	95%CI	aHR	95%CI	aHR	95%CI
SO_2_ (ppb)	Q1, < 2.63						
	Q2, 2.63–3.21	1.40	(1.28, 1.54)	1.57	(1.36, 1.81)	1.30	(1.16, 1.46)
	Q3, 3.21–3.54	1.05	(0.96, 1.15)	1.04	(0.89, 1.21)	1.06	(0.94, 1.19)
	Q4, > 3.54	8.43	(7.84, 9.07)	8.69	(7.72, 9.77)	8.28	(7.55, 9.09)
CO (ppm)	Q1, < 0.37						
	Q2, 0.37–0.45	1.33	(1.23, 1.44)	1.35	(1.19, 1.53)	1.31	(1.18, 1.44)
	Q3, 0.45–0.55	2.08	(1.93, 2.24)	2.18	(1.94, 2.45)	2.01	(1.82, 2.21)
	Q4, > 0.55	3.03	(2.83, 3.25)	3.28	(2.94, 3.65)	2.87	(2.63, 3.14)
NO (ppm)	Q1, < 2.56						
	Q2, 2.56–4.05	1.53	(1.41, 1.66)	1.62	(1.42, 1.84)	1.48	(1.34, 1.64)
	Q3, 4.05–6.12	1.92	(1.77, 2.08)	2.11	(1.86, 2.40)	1.80	(1.62, 1.99)
	Q4, > 6.12	3.47	(3.23, 3.73)	3.84	(3.43, 4.31)	3.24	(2.96, 3.55)
NO_2_ (ppm)	Q1, < 16.1						
	Q2, 16.1–21.2	1.51	(1.40, 1.63)	1.64	(1.46, 1.85)	1.43	(1.30, 1.57)
	Q3, 21.2–26.9	1.12	(1.04, 1.21)	1.28	(1.14, 1.45)	1.02	(0.92, 1.12)
	Q4, > 26.9	3.72	(3.48, 3.98)	4.15	(3.73, 4.62)	3.46	(3.18, 3.77)
PM_2.5_ (µg/m^3^)	Q1, < 17.0						
	Q2, 17.0–18.4	0.18	(0.13, 0.26)	0.20	(0.12, 0.35)	0.17	(0.11, 0.26)
	Q3, 18.4–26.3	4.82	(4.29, 5.42)	5.33	(4.41, 6.45)	4.52	(3.90, 5.23)
	Q4, > 26.3	21.9	(19.7, 24.5)	23.0	(19.2, 27.6)	21.3	(18.6, 24.5)
PM_10_ (µg/m^3^)	Q1, < 34.8						
	Q2, 34.8–39.9	0.95	(0.82, 1.10)	0.99	(0.78, 1.26)	0.92	(0.77, 1.11)
	Q3, 39.9–49.7	5.94	(5.33, 6.63)	6.58	(5.50, 7.86)	5.56	(4.84, 6.38)
	Q4, > 49.7	15.6	(14.1, 17.4)	16.2	(13.7, 19.3)	15.3	(13.4, 17.4)

aHR, adjusted hazard ratio for age, sex, urbanization level, and comorbidities such as alcohol abuse/dependence, tobacco abuse/dependence, chronic obstructive pulmonary disease, and asthma; CI, confidence interval; CO, carbon monoxide; NO, nitric oxide; NO_2_, nitrogen dioxide; PM, particulate matter; Q, quartile; SO_2_, sulfur dioxide

## Discussion

We conducted a nationwide retrospective study with approximately 12-year follow-up data from 286,044 Taiwanese residents to evaluate the association between sarcopenia and air pollutant exposure, and found a significantly increased risk of sarcopenia in both male and female residents exposed to high levels of air pollutants.

There is growing evidence that air pollution exposure increases inflammatory markers, such as C-reactive protein (CRP); interleukins (ILs)-1, 6, 8, 10; tumor necrosis factor (TNF)-α; vascular cell adhesion molecule-1 (VCAM-1); intercellular adhesion molecule-1 (ICAM-1); reactive oxygen species (ROS); and reactive nitrogen species (RNS) [36–43]. CRP was independently associated with muscle strength impairment [44]. Furthermore, elevated concentrations of TNF-α, IL-6 and CRP are frequently identified in sarcopenia patients [45, 46] These cytokines probably trigger the activation of the ubiquitin-protease system and develop sarcopenia [47, 48].

Aging has been shown to decrease skeletal muscle mass and strength via oxidative stress and molecular inflammation in both human and animal studies [20, 21, 49]. During the aging process, the balance between degradation and resynthesis of skeletal muscle proteins is disrupted [50]. The results of a community-based study suggested that increased oxidative stress is significantly associated with grip strength in older women [20]. In addition, patients with sarcopenia have a significantly higher erythrocyte sedimentation rate (ESR) and CRP levels than controls, [19] and ROS/RNS plays an important role in the development of sarcopenia [51].

Air pollution sources include traffic emissions, fossil fuel combustion, and emissions from industries, agriculture, and power plants [52, 53]. The degree of air pollution is highly associated with urbanization [54]. We obtained air pollutant levels from 74 AQMS across all over Taiwan. Previous findings suggest that air quality in northern Taiwan has seen improvement over the last decade. Nonetheless, central and southern Taiwan have not experienced significant advancements in air quality. In these regions, PM concentrations have consistently exceeded the EPA’s established exposure standards, necessitating measures to mitigate their adverse effects on public health [55]. The study results show that the PM has the greatest impact on sarcopenia risk in residents living in central and southern regions.

Ethanol exposure impairs skeletal muscle protein synthesis and induces muscle autophagy [13, 56, 57]. However, previous studies have not found a significant association between alcohol consumption and sarcopenia [28–30]. Cigarette smoking is a common risk factor for many diseases including sarcopenia, as it causes oxidative stress and chronic inflammation [31–34]. COPD is a chronic inflammatory disease that affects the airways, [58, 59] showing similar symptoms to asthma. Both immune responses and chronic inflammatory diseases affect the airways [60, 61]. Sarcopenia frequently co-occurs with COPD or asthma, [62–65] similar to its relationship with osteoporosis [66–68]. In addition, several studies have shown that visceral fat with abundant inflammatory cells is the primary source of inflammatory cytokines, such as IL-1β, IL-6, and TNF-β [69–71]. Considering the above, we performed a sex-stratified multivariate Cox regression, adjusting for urbanization, asthma, obesity, osteoporosis, and estrogen supplementation.

This was a well-designed cohort study with a long follow-up period adjusted for various confounding factors. However, this study had some limitations. First, a surveillance bias might have been present. Level of urbanization might have been associated with medical convenience, resulting in different prevalence of sarcopenia in urban and rural areas. The Taiwanese government has established a single-payer compulsory social insurance system covering > 99% of residents, which balances medical convenience between urban and rural areas by providing free medical care [72, 73]. Second, living area was defined by the location of the institute where residents sought medical care during the study period. However, residents who had no medical records during this period were not enrolled; further, they tended to live in areas with low levels of air pollutants, resulting in an underestimated risk of sarcopenia. Third, although indoor air pollutant levels are associated with building characteristics, [74] their concentration could not be evaluated in this nationwide study. However, there is no evidence that buildings differ in places with different levels of air pollution in Taiwan, or that they cannot block outdoor air pollutants [75, 76]. Nevertheless, additional studies are required to clarify the impact of indoor air quality. Fourth, the major limitations of the NHIRD study are lack of healthy behavior and dietary habits. Diagnostic criteria for alcohol abuse/dependence were based on drinking behaviors and patients’ attitudes. In previous NHIRD studies, COPD, asthma, and tobacco abuse/dependence were considered instead of smoking status [77–79]. Thus, we consider COPD, asthma, tobacco abuse/dependence and alcohol abuse/dependence as the proxy variables for smoking status and alcohol consumption. Fifth, air pollution levels in the residential areas of NHIRD insurants were assessed based on the nearest air quality monitoring stations to clinics or hospitals. This approach may introduce bias in the results, as the measured air quality and urbanization levels could deviate from the true values, particularly when participants have lengthy commutes between their residences and medical facilities. Therefore, additional personal air sampling is necessary to validate these observations.

In summary, this study discovered a significant positive relationship between air pollution exposure and development of sarcopenia in both men and women. According to this finding, less polluting residential areas are required in older societies to slow the progression of chronic diseases like sarcopenia. However, further clinical and experimental studies are required to confirm these findings.

## Data Availability

The data that support the findings of this study are available from Taiwan Ministry of Health and Welfare (MOHW), but restrictions apply to the availability of these data, which were used under license for the current study, and so are not publicly available. Data are however available from the authors upon reasonable request and with permission of the Taiwan MOHW.
